# Social Science and Neuroscience beyond Interdisciplinarity: Experimental Entanglements

**DOI:** 10.1177/0263276414537319

**Published:** 2015-01

**Authors:** Des Fitzgerald, Felicity Callard

**Affiliations:** King’s College London; Durham University

**Keywords:** biology, collaboration, critical neuroscience, critique, experiment, methodology, the social

## Abstract

This article is an account of the dynamics of interaction across the social sciences and neurosciences. Against an arid rhetoric of ‘interdisciplinarity’, it calls for a more expansive imaginary of what *experiment* – as practice and ethos – might offer in this space. Arguing that opportunities for collaboration between social scientists and neuroscientists need to be taken seriously, the article situates itself against existing conceptualizations of these dynamics, grouping them under three rubrics: ‘critique’, ‘ebullience’ and ‘interaction’. Despite their differences, each insists on a distinction between sociocultural and neurobiological knowledge, or does not show how a more entangled field might be realized. The article links this absence to the ‘regime of the inter-’, an ethic of interdisciplinarity that guides interaction *between* disciplines on the understanding of their pre-existing separateness. The argument of the paper is thus twofold: (1) that, contra the ‘regime of the inter-’, it is no longer practicable to maintain a hygienic separation between sociocultural webs and neurobiological architecture; (2) that the cognitive neuroscientific experiment, as a space of epistemological and ontological excess, offers an opportunity to researchers, from all disciplines, to explore and register this realization.

## Introduction

A spectre haunts social science: the spectre of the brain. We are, writes the historian Roger Cooter, in the midst of a pernicious ‘neuro-turn’, in which scholars assume that, among other things, ‘“the social,” and “life” itself have … undergone a refashioning as a result of the new life sciences in general and neurobiology in particular’ (2014: 146). With the advent of this turn, the anthropologist Emily Martin argues:

we are seeing the effects of a form of reduction that is likely to impoverish the richness of human social life .… Social practices involved in gift giving, child raising, courting, working, cohabiting, co-organizing and a myriad others – all situated in particular contexts, times and places – fall out of the picture and do not return. (2010: 369)

Such laments – and they are not idiosyncratic (Ortega and Vidal, 2007; Choudhury et al., 2009) – should be situated within a broader anxiety, evident in the humanities and social sciences in the last decade, about the increasing tendency for researchers, from several disciplines, to blur the boundaries between the traditional concerns of a social or humanistic interest, and the technologies and methods of the neurosciences. Many interpretative and humanistic scholars have thus begun to sense, within their once-secure intellectual domains, the soft, ominous tread of the new brain sciences (Cromby et al., 2011). And if this intellectual development is truly a ‘refashioning of our older disciplinary habits of the heart’, Cooter continues, in an unusually dramatic intervention, then there can be ‘no task … more vital and urgent’ than its critique (2014: 154).

Perhaps we should not be too surprised by such talk: ‘the materiality of the world’, Helga Nowotny reminds us, has a tendency to ‘upset the existing intellectual division of labour, and the cognitive and practical order upon which boundaries rest’ (2005: 24). But if we are indeed living in a neurobiological age, what are we actually to do – and we use ‘we’ here performatively, to gather together social theorists, humanists, and qualitative social scientists – when the webs of human social and cultural life that we had come to understand as *our* particular object of knowledge seem more and more open to being figured neuroscientifically and experimentally?^1^ Certainly, solutions have been offered – that we subject the new brain sciences to a refined socio-critique (Ortega and Vidal, 2007); that we demand their political reform (Choudhury et al., 2009); that we welcome them into cultural theory (Wilson, 2004a, 2011); that we use them to upset our taken-for-granted assumptions (Stafford, 2008); that we embed them within our accounts of the political (Connolly, 2002); that we regard their deconstruction of subjectivity as more effective than Derrida’s own (Malabou in Johnston and Malabou, 2013); that we join them (Roepstorff et al., 2010); that we analyse them (Dumit, 2004; Cohn, 2008); that we reject them (Martin, 2004); that we accept them (Franks, 2010); that, taking the longer view, we locate them within a much thicker braid of social and biological torsion (Rose, 2013). So, on and on, go the debates.

We are in various states of agreement and disagreement with these proposals. Recent calls by Rose (2013) and by Rose and Abi-Rached (2013) for new ways of figuring the space between the social- and neuro-sciences have been particularly important for what follows. But we want also to expand that discussion into a new terrain: the terrain of the experimental. If there has been extensive discussion of what these developments entail conceptually and institutionally for the social sciences and humanities (Cromby, 2007; Pickersgill, 2013), there has been less critical attention given to what the rise of the neurobiological age might entail for the social sciences and humanities *methodologically* and *in practice*. If a wider social-science literature is taken up with expressions of straightforward gratitude for, or equally straightforward rejection of, findings from neuroscientific experiments, there has been little suggestion that *experimental labour itself* might be worthy of sustained attention from social scientists and humanists.^2^

What would happen if we changed the spatio-temporal dynamics of this scene? What if social scientists and humanists moved away from conceiving the domains of the neuroscientific and the experimental as the unchallenged province of the brain sciences – whose apparent territorial expansiveness they must welcome, ignore or repel? Could the neuroscientific *experiment*, as a rich and ambiguous way of producing different knowledges, help us to think some more creative and entangled ways of exploring these questions? In this article, we claim another intellectual space, cutting across the contemporary neurosciences and social sciences. There are three elements to our proposal: (1) If there is now much critical, conceptual discussion about the space ‘between’ the social- and neuro-sciences, there is strikingly little attention to how *methodological* novelty, serendipity and contingency might conjure a more constructive space of shared collaboration. (2) A turn to ‘experiment’ offers an entry-point to this space. We fix on experiment because it captures both: (i) the means by which cognitive neuroscience derives many of its epistemological claims from laboratory practices, and (ii) a wider ethos of openness to different procedures of action and investigation (Morawski, 1988). And if we are preoccupied with cognitive neuroscientific experiments that employ magnetic resonance imaging (MRI) (Poldrack, 2010; Bandettini, 2012), our proposal extends an invitation to other histories and territories of ‘experimental entanglement’ – to correlational or observational studies, to clinical spaces, to behavioural research, or indeed to any other site of the broadly-conceived experimental repertoire of the new brain sciences. (3) Through our turn to methodological novelty, and to experiment in all its guises, we propose what we believe to be a more compelling platform for scholars who may have some urge, now, to think through the intersections of neurobiological and social life. In particular, we want to help such scholars circumvent a burgeoning, but bloodless and sterile, literature on ‘interdisciplinarity’ *between* the social sciences and the life sciences. ‘Experimental entanglements’ is our name both for a new way of addressing these questions *and* for the contingent, unstable, fleeting empirical commitments in which that argument is embedded.

In what follows, we do not follow these threads in order, but work them through a four-part argument. First, we consider recent developments in the neurosciences, and we show how collaborative possibilities for the ‘social’ sciences have opened up around them. Second, we offer a sustained analysis of literatures that have already interpreted this space, which we group under three headings: *critique*, *ebullience* and *interaction*. Third, we argue that this entire discursive space is torqued by a series of epistemological and ontological commitments that limit the scope of collaboration between the neurosciences and social sciences. We name this limitation ‘the regime of the inter-’. Fourth, we elaborate our own programme of ‘experimental entanglements’, and we argue that our interest in contingent, fleeting moments of methodological novelty may offer potent possibilities for inhabiting the space we have identified. At the heart of the article is an argument for re-thinking the laboratory-based experimental domains of the cognitive neurosciences as both spaces and moments for firing strange alliances between neuroscientists and social scientists.

In a related publication (Callard and Fitzgerald, under contract), we offer pragmatic advice on interdisciplinary interaction for collaborators from all disciplines. But this article has a narrower remit: here, we intervene in *internal* discussions, within the social sciences and humanities, about possibilities for, and encouragement towards, collaboration with the neurosciences. Our interest is in significantly expanding that conversation: the article is aimed at scholars within those disciplines who have some urge towards concrete engagement with the neurosciences, but who remain unmoved by today’s arid rhetoric of ‘interdisciplinarity’. The unabashedly programmatic aim of this article is to put pressure on the usual ways in which such possibilities between the social sciences and the neurosciences are understood (e.g. European Commission, 2011). Our article sets out the core conceptual ground for the elaboration of an alternative programme, paying particular attention to the ‘experiment’ as a space of intervention, and using ‘entanglement’ explicitly to depart from logics of ‘engagement’ and ‘dialogue’.^3^

## Why the Neurosciences?

Today, cognitive neuroscience^4^ is frequently held up as the greatest intellectual resource for the humanities and social sciences (Pinker, 2013) – or the gravest intellectual threat (Tallis, 2011). This prominence is inseparable from the neuroscientific claim on the ‘space inside the skull’ (Beaulieu, 2000), that prized locus of so much interpretative scholarship. If there is still much research to be done on the uneven historical and geographical contours of neuroscientific authority, it remains undeniable that many facets of human life that were, for much of the 20th century, primarily understood through the abstractions of ‘culture’ or ‘society’ – commercial and economic life, governance, historical change, identity, distress and suffering – are increasingly understood as functions of the cerebral architecture of individuals or of groups of individuals (for examples, see Adolphs, 2003; Camerer et al., 2005; Chiao, 2009; for reflections, see Rose, 2010; Vrecko, 2010; Matusall, 2012).

There are many ways to respond to this social fact. We start, here, from the realization that in a growing number of research areas, bioscientists, as Nikolas Rose maintains, increasingly characterize

living organisms as dynamic and complex systems, located in a dimension of temporality and development, and constitutively open to their milieu – a milieu that ranges in scale from the intracellular to psychological, biographical, social and cultural. (2013: 5)

Indeed, and especially within the new brain sciences, it is clear that, just as technologies have emerged to measure the workings of the central nervous system *in vivo*, so is that system becoming conceptually inseparable from the social, cultural and familial contexts in which it developed: biology, Maurizio Meloni points out, ‘has become porous to social and even cultural signals to an unprecedented extent’ (2014: 2; cf. Bird, 2007; Hyman, 2009; Niewöhner, 2011). On the one hand, of course, this presents a significant opportunity for social scientists. As a recent *Nature* editorial pointed out:

Sociologists have been studying human environments for decades, and have tallied the social damage that stresses such as poverty or child abuse can cause. Biologists are now in a position to benefit from their insights. (*Nature*, 2012: 143)

If we are not bowled over by this description of what sociological labour might offer, it seems indisputable that there is something important that social scientists now ‘offer’ the life sciences. As Ilina Singh points out, the ‘emerging disintegration of the nature-nurture divide’ from within the biosciences offers a new collaborative space for social scientists (2012: 316–17). And the neurosciences, especially, Rose and Abi-Rached remind us, are currently ‘struggling towards a way of thinking in which our corporeality is in constant transaction with its milieu’ (2013: 3). We are in significant agreement with both the general claim that social science has something to offer and that new forms of collaboration should be risked in order to grasp these opportunities. But here we append two further remarks.

First, there is a risk of these careful arguments being (mis)interpreted as encouragement to leap faithfully into a newly socialized biology. But we are painfully aware that the ‘social’ of a ‘social neuroscience’ is often a rather mangy-looking beast – an animal quite alien to the rich and fat understanding of a century-old anthropology or sociology (Matusall, 2012). We also worry about how ‘culture’ is commonly imagined as just another input within a straightforwardly bioscientific schema, and we know well that awkward questions remain about the epistemological politics at stake within these generous-looking invitations (Choudhury and Kirmayer, 2009; Young, 2012). We cannot ignore, as scholars trying to make a space for our interests within a shrinking, instrumentalizing academy, the shifts in scholarly prestige that surely guide, for example, the increasingly-warm *rapprochement* between analytic philosophy and cognitive neuroscience (e.g. Smith, 2012). We have squirmed our way through too many ‘interdisciplinary’ meetings to remain innocent of just how narrowly the world outside the skull sometimes gets figured within these ‘biosocial’ narratives.

Second, and this is where our article finds much of its impetus, it has not been easy to imagine or specify how these collaborations might be enacted in practice. This is an intrinsically vexed question, and we offer no simple solution here. As we will argue below, however, one way to move the discussion forward is to think more creatively about *experiments*. While we have been inspired by broad calls for social scientists to take up new possibilities for collaboration, we have often been dismayed by the narrow rhetorics and frameworks of interdisciplinarity that seem to govern actual, real collaborative spaces *beyond* those calls. And yet, at the same time, our collaborative imaginaries have consistently been fired by experimental moments – admittedly often short, contingent, serendipitous – that we have painstakingly sought, located and nurtured within such spaces. There *are*, now, real opportunities for collaboration between the social sciences and neurosciences. But these opportunities are often occluded by the narrow discursive range of contemporary ‘interdisciplinarity’. This article therefore draws attention to some more *experimental* modes for re-imagining that space. Before we elaborate on our own approach, we first distinguish it from the most prominent modes through which the relationship between the social sciences and the neurosciences has hitherto been understood.^5^ In line with our programmatic aim, we have distilled the core features of heterogeneous and expansive endeavours. We trust that the benefits of clarity outweigh the risks of caricature.

## Three Modes of Neuro-Engagement

### Critique^*6*^

Arguably the most common way of positioning the social sciences and humanities in relation to cognitive neuroscience is to interpret their task as the critique of neurobiological chauvinism. This mode uses the tools of historical, social and cultural analysis as external methods to either: (1) uncover unconscious or hidden biases within the new brain sciences, and to locate nefarious social, political, economic and epistemic agendas within them (e.g. Ortega and Vidal, 2007; Choudhury et al., 2009); or (2) deflate particular neuroscientific trends or claims that have found favour within the humanities or social sciences (e.g. Ashton, 2011; Kramnick, 2011).^7^

These engagements commonly lean on longstanding claims concerning the fundamentally *sociocultural* nature of scientific (including neuroscientific) knowledge (Pickering, 1992). The most compelling articulations of this critique come from a trio of scholars and groups who have argued, trenchantly, for the fundamentally sociocultural basis of the neuro-reductionist urge (Martin, 2004, 2013), the political ill-effects of this urge on our senses of self (Ortega and Vidal, 2007; Vidal, 2009), and the need for the new brain sciences to be radically re-imagined (Choudhury et al., 2009; Slaby and Choudhury, 2012). Emily Martin was perhaps the first to identify the emergence of a cultural figure whose levels ‘begin with molecules, but go no farther than the central nervous system’ (2000: 574). Thus, Martin argues, ‘all of what anthropologists call culture has drained through the hole and dissolved in the realm of neural networks’ (2000: 576). Martin locates the cultural and institutional desire for the ‘restraining force’ of this ‘ahistorical concrete body’ in manifestly social developments: for example, in the need for a reaction to the mania and wildness of *fin de siècle* capitalism (2000: 576, 581), in psychiatric-expert attempts to ‘snare’ the ‘criteria of rationality’ and the ‘meaning of language’ (2004: 194) and in ‘contempt for anything that limits the kind of commensurability that our markets and systems of governance demand’ (2013: s157).

But there is a deeper point embedded here, and this is Martin’s argument for the ontological primacy of the sociocultural over the neurobiological, in order to ‘detect the real prejudices hidden behind the appearance of objective statements’ (Latour, 2004: 227). Fernando Vidal, similarly, has argued that attempts to locate some organic and naturalized account of the self in fact long precede the emergence of the new brain sciences – that this is an ideology on to which neurobiology is mapped *post hoc*: ‘the idea that “we are our brains” is not a corollary of neuroscientific advances, but a prerequisite of neuroscientific investigation’ (2009: 7). A related argument has been made by the exponents of ‘critical neuroscience’ (Choudhury et al., 2009; Choudhury and Slaby, 2012). The essence of this account, which is inspired by the Frankfurt School,^8^ is not to tear down neuroscience but to inculcate among neuroscientists:

self-critical practices, which aim to achieve reflective awareness of the standpoint-specific biases and constraints that enter into the production, interpretive framing and subsequent application of neuroscientific knowledge. (Choudhury et al., 2009: 65)

In other words, neuroscience *itself* should be reformed as a critical practice, and become aware of its own political and economic standpoints. But neuroscience must *also* harness the ‘emancipatory potential’ for neuroscientific workers to reflexively labour upon the biases embedded in their own practices (2009: 65). Once again, the point is to understand ‘neuroscience itself as a cultural activity’ – to re-situate it within a ‘social structure’ and re-formulate it as a practice run through with economic drivers, political climates and cultural contexts (2009: 62–4).

Such critiques are salutary reminders of the need to devote analytical attention to the ‘logic of the neuroindustry’ – and there are resonances between our proposal and some of the more pragmatic steps proposed by scholars in this tradition (Slaby and Choudhury, 2012). But the stance of critique tends too readily to wield the master term ‘reductionistic’ to characterize both neuroscience’s own knowledges *and* its effect on other disciplines (e.g. Kirmayer and Gold, 2012). In fact, an insistence on ‘reduction’ renders much of what is most analytically interesting about neuroscience – including its relationship to other domains, and how those relationships might be re-imagined – invisible. One central example comprises the fascinating and novels ways in which ‘culture’ and ‘neurobiology’ are drawn *together*, and how bodies and cultures have become experimentally legible *in* one another (e.g. Lende and Downey, 2012: 23). In fact, relations between metabolic brain processes, sociocultural environments and ‘mental processes’ are being repeatedly experimentally *re-adjudicated* in cognitive neuroscience. And this is just one instance of the uneven and creative ways in which the dynamic relationship ‘between’ – though that is not quite the right adjective – the ‘neurobiological’ and the ‘cultural’ is kept in play (Callard and Margulies, 2011).

Where we most significantly depart from colleagues in the critical tradition is in our refusal to cede ontological primacy to the *sociocultural* within this terrain, certainly in light of the far-reaching theoretical challenges that have been launched at such a premise (Whitehead, 1964; Haraway, 1991; Braidotti, 2006). Interestingly, the critical literature often perfectly well sees, but then usually scotomizes, the complexity and subtlety of the new brain sciences – missing, in particular, how they think through, and work on, the tangled imbrication of bodies, brains, minds, subjectivities, lives and machines. Kelly Joyce, for example, draws on a powerful image from Elizabeth Grosz (1994) to suggest that MRI images ‘“etch together” local decisions and priorities, technology, and aspects of the physical body to produce what is perceived as cutting-edge, authoritative knowledge’ (Joyce, 2008: 70). But what gets missed in Joyce’s desire to show that ‘there is nothing natural or inevitable’ about MRI is precisely the intellectual force of a science that *can* ‘etch together’ local politics, de-oxygenated blood, sick bodies, nuclear physics, and the clinical gaze to produce what for many is a convincing image of a person, and a body (2008: 20).

### Ebullience

If much neuro-critique is built on a presumption of the ontological primacy of ‘culture’, then the ‘ebullient’ mode tends to take experimental results and theoretical statements from the neurosciences as more-or-less true – with little contest or context, and in the absence of a sense of the wider, often fierce, epistemological and ontological debates *within* those sciences. As Papoulias and Callard (2010) have argued, the emergence of what is commonly now known as ‘affect theory’ within cultural studies has often been the ground for such enthusiasm. Here, many social and cultural theorists rest accounts of the dynamic inter-relations between cultural theory and neuroscientific fact via skilled and lengthy attention to the former – and surprisingly thin, often naïve, summaries of the latter.

Strikingly, many ebullient engagements with the neurosciences from humanists and social scientists barely stray further than scientists’ ‘crossover’ publications for lay audiences (here, Damasio’s volumes (2000, 2004, 2006) are highly favoured) – evidence of the strangely credulous and limited reading practices of those who accrue intellectual capital *precisely for* the acuity and breadth of their reading. The philosopher Catherine Malabou, for example, has provided one of the most provocative and renowned accounts of how current research in the life sciences (and particularly the neurosciences) pushes beyond post-Husserlian conceptualizations of subjectivity in Continental philosophy (2008, 2012; Johnston and Malabou, 2013). Central to Malabou’s argument is her conviction that current neurobiology effects wide-ranging transformations in understandings of affect, producing a more radical challenge to conceptualizations of subjectivity than those articulated by deconstruction and psychoanalysis: ‘Current neurobiology is engaged in a deep redefinition of emotional life’, Malabou argues:

The brain, far from being a nonsensuous organ, devoted solely to logical and cognitive processes, now appears … to be the center of a new *libidinal economy *.… A new conception of affects is undoubtedly emerging. (Johnston and Malabou, 2013: 3)

But this authoritative characterization concerning the huge and heterogeneous field of neurobiology is founded almost entirely on Malabou’s enthusiastic reading of a very select number of scientists who have published for a general audience. And while Malabou’s monograph *The New Wounded* (2012) is full of acute and contrapuntal readings of Freud, her engagements with the neurosciences are largely restricted to adulatory reiterations of sentences from Antonio Damasio, Joseph LeDoux and Oliver Sacks. In developing our own formulations about our relations with the neurosciences, we have gained much from the audacity of Malabou’s forays. But what we miss in her publications is a strong sense of scientific nuance and breadth: Malabou’s monographs demonstrate limited engagement with peer-reviewed scientific publications, with internal criticisms of Damasio, and with histories of science – any one of which might provide a thicker, more adhesive texture for claims regarding a field’s ‘deep redefinitions’ and the challenges these pose to theorizations from the humanities.

Or consider Brian Massumi’s influential essay, ‘The Autonomy of Affect’, which aimed to provincialize a reliance on signification and language in cultural theory by drawing attention to the ‘dynamism’ of the neurological sciences (1996: 100). As we have ourselves become more intimately involved with experimental spaces, it strikes us that the neuroscience that emerges through Massumi’s account is, in contrast, not at all dynamic, or flexible, or even very interesting. Neuroscience is in fact figured by Massumi as lumpen, univocal, and tediously certain. Moreover, the science on which Massumi’s theoretical claims rests makes startlingly brief appearances – accurately characterized by Ruth Leys as a ‘strategic’ and ‘fleeting’ service for Massumi’s ‘rather opaque philosophical-speculative reflections’ (Leys, 2011).

‘The manner in which “science” is often invoked in cultural theory texts’, Papoulias and Callard point out, ‘testiﬁes to a desire for a certain kind of revelation that science will be able to satisfy’ (2010: 36–7; see also Barnett, 2008). Authors in the ebullient tradition, in their desire to designate generative spaces for the mingling of biology and culture, unintentionally *foreclose* the space for a dynamic and mutually constitutive traffic across them; they are much too willing to assign to the natural and experimental sciences the task of generating the findings that will confirm, verify and/or reveal the theoretical insights of cultural and social theory. If this mode of engagement with neuroscience is characterized by ebullience towards its desired objects and partners, it tends to remain demurely secluded from the hubbub of experimentation itself.

### Interaction

A relatively small group of scholars has, in recent years, begun to undertake the rather thankless task of locating a conceptual space between the social sciences and the neurosciences – while resisting the attention-grabbing rhetorics of critique or ebullience. What we term the ‘interactive mode’ is characterized neither by a desire to provincialize the pretensions of the neurosciences nor by an uncritical acceptance of insights from those spaces. Instead, scholars focus on research on humans’ neurological propensities but, crucially, they also maintain an epistemic parity between this research and the traditions and paradigms of the interpretative and social sciences. These works grant the same kind of sustained and critical attention to neurology and neurobiology as they do to the interpretative social sciences. They read, in the neurosciences, a complementary desire for mutuality, and a willingness to allow insights from sociocultural theory to fold back onto neuroscientific research; in so doing they strive for a neurobiology that might help to develop different kinds of theories about the contemporary figure of the human *as such*.

In *Neuro*, for example, Nikolas Rose and Joelle Abi-Rached argue that new styles of thought emergent in neuroscience:

offer the possibilities of a more positive role for the human and social sciences, an opportunity to seize on the new openness provided by conceptions of the neuromolecular, plastic, and social brain, and to move beyond critique and find some rapprochement. (2013: 24)

Such a rapprochement, they argue, may even contribute to a new kind of progressive thought – refusing an account of human societies as composed of maximizing, individual organisms, or of governmental modes designed to regulate such organisms (2013: 234). ‘At their most sophisticated’, Rose and Abi-Rached suggest, ‘[the neurosciences] are struggling towards a way of thinking in which our corporeality is in constant transaction with its milieu, and the biological and the social are not distinct but inter-twined’ (2013: 3). Other scholars in the interactive mode have tried to mobilize such transactions: Andreas Roepstorff (2001), for example, has used his dual identity as a brain-imager and a cultural anthropologist to revive the animalistic, world-experiencing ‘biophilosophy’ of Jacob von Uexküll, and has argued (Roepstorff et al., 2010) that re-thinking forms of social interaction as ‘patterned practices’ might operationalize the entanglement of cultural and neural networks.^9^

Another sustained attempt to re-calibrate relations between the neural and the sociocultural has been made by Elizabeth Wilson (1998, 2004a, 2004b, 2010, 2011), whose broad project works the neurological into feminist accounts of the body, and to feminist theory more generally. In tandem with other accounts that have mobilized scientific literatures to explore and conceptualize affective relationality (Sedgwick, 2003; Blackman, 2008), Wilson’s cultural-theoretical project pursues a mingling with neurology in terms of the ‘potential in the neurosciences for reinvention and transformation’ (2004a: 13). She argues that, between psychology and neurology, ‘forces of influence and determination are more mutually entangled than the critics of neurological determinism have hitherto acknowledged’ (2004a: 16). In the circuit of body, psyche, and environment, we do not find a relationship of simple causation but rather ‘a system of mutual constitution from which no particular element emerges as the originary, predetermining term’ (2004a: 19). Thus: ‘neurological material is more confident, flexible, resilient, and assertive than many critics have yet acknowledged’ (2004a: 22). This, on Wilson’s account, is what socio-critique prevents us from seeing: ‘by disconnecting biology from its constitutive relations with other ontological systems’, she argues, ‘biology becomes isolated and destitute’ (2004a: 70).

Our project of experimental entanglement is indebted to this stance. Following Rose, we are in pursuit of ‘an affirmative relationship’ with an emerging ‘new and non-reductive biology of human beings and other organisms in their milieu, … which can thus be brought into conversation with … the social and human sciences’ (2013: 24). With Wilson, we seek a neuroscience that ‘may … be a resource for theoretical endeavour, rather than the dangerous and inert substance against which criticism launches itself’ (Wilson, 2004a: 29; cf. Stafford in Turnbull, 2007: 347). The work that remains, then, is to think about how such insights can be realized in empirical projects, or how they can be more concretely situated within a more expansive research practice. While we are hardly the first to pursue this question, our experience is that when similar programmes *are* moved onto a more empirical terrain, the core insights of the interactionist mode have been hard to maintain. Too frequently, soft boundaries between social and neural are maintained through a model of disciplinary partnership (e.g. Lende and Downey, 2012); the biosocial nexus starts to look distinctly bio-centric (e.g. Chiao, 2009); the empirical project distances itself from (and thus struggles to move) the core concerns of sociocultural knowledge (e.g. Roepstorff and Frith, 2012); or the disciplinary ‘role’ that each intellectual party plays in the programme becomes solidified, such that the possibilities for folding insights *across* epistemological domains are reduced (e.g. Sambo et al., 2010). We see a gap, then, in which the final step is not yet enacted in practice or where there tends to be a limited working-through of the dynamic complexity of the ontological and epistemological reshufflings that might be enacted *through* such practice. It might, indeed, be such an absence that has allowed the more critical and ebullient voices to dominate the debate within the social sciences.

## The Regime of the Inter-

The modes of ‘critique’ and of ‘ebullience’ seem to sit at opposite ends of the spectrum. But we suggest that they are animated by a shared commitment – namely, that the sociocultural and the neural are different domains of knowledge, and that they address themselves to different kinds of objects, or to different aspects of objects. For the critic, a commitment to this divide between the sociocultural and the neural means defending the boundary-points, and re-asserting the strict differences between the two areas.^10^ For the enthusiast, the divide describes instead a hierarchized division of labour – and a willingness to render unto the neurosciences what is truly neuroscientific. But if the critic and the enthusiast are very different from one another, they share the most important commitment: namely, that there are things, and ways of knowing things, that are sociocultural; and there are things, and ways of knowing things, that are not. The only difference is that the critic insists that this is how it should be, whereas the enthusiast would rather redraw where the line falls, in acquiescence to new neuroscientific knowledge about (what were previously thought of as) sociocultural preoccupations. But this is a trivial distinction. The existence and salience of what is really important here – the dividing-line itself – is never in question. Slaby and Choudhury, for example, place ‘particular emphasis on the social’ in the face of a fashionable and shallow ‘ontological hybridization’ (2012: 36–7). Von Scheve, by contrast, calls on sociologists to attend to ‘actual neuroscientific findings’ (2012: 256). But for each of them, there is a thing called social science that addresses itself to one kind of object; and there is a thing called neuroscience that addresses itself to another. The only controversy is about whether current flirtations between the two should be consummated. This debate thus operates entirely within an unquestioned, shared space, which we call ‘the regime of the inter-’.

The ‘regime of the inter-’ refers all analysis about the space between the social sciences and the neurosciences to a guiding question: given that there is the possibility of overlapping interests and objects between these sciences, then how large should that space of overlap be, how should it be populated, what kinds of objects should be located within it, and what should count as a sufficiently ecumenical research programme to address those objects? But this regime excludes consideration of the history, topology, and salience of that space *as such*; about the border-practices that bind it; and about how even the very conjunction ‘between’ forecloses other ways of conceptualizing its characteristics, and the relationalities comprising it. Moreover, we contend that this regime governs most – if not all – of the institutional spaces that lay claim on what is seen as the growing need for *inter*disciplinary labour *between* the neurobiological sciences, and the social sciences and humanities.^11^ Our intimacy over a number of years with a number of these explicitly designated ‘interdisciplinary’ spaces has strengthened our conviction that their governing ethic of epistemological seclusion (of the social sciences/humanities from the neurosciences, and vice versa) is a recalcitrant fantasy – one premised on a sanitized history of disciplinary domains, of the frequent intimacies that have enjoined them, and of their respective objects of study (for alternative genealogies, see Donzelot, 1988; Renwick, 2012; Rose, 2013). In this regime, certain visions of territory – along with the corollary concepts of borders, incursions, and empire-building – tend to loom large. In contrast, our proposal takes for granted the conceptual, methodological and terminological crossings – admittedly often forgotten, often fugitive – that have long tacked back-and-forth between (and within) the domains of the sociocultural, the psychological and the neural, and that have been variously distributed within and across so-called ‘disciplinary divides’. We think, for example, of the genetic (and eugenic) history of early British social science (Osborne and Rose, 2008), of the presence of non-human animals in a developing sociology (Shearmur, 2013), or of the deeply uncanny biology bound within long strands of 20th-century psychoanalysis (Laplanche, 1989). Our interest, as both subjects and analysts of an emerging neurobiological age, lies in understanding how social scientists might best employ and re-energize that rich archive of crossings. We want to know how they – we! – might forge different and unexpected relations, whether intellectual, methodological, or affective, with the neurosciences.

Our proposal thus sets itself against the ‘regime of the inter-’. ‘Experimental entanglements’ start *in media res*, where there are neither neatly bordered disciplines nor any clear dispensation regarding which ‘objects’ of study are appropriate for each. Our gambit is that if a different sociocultural research practice – one that attempts to do epistemic and ontological justice to the fertile crossings between the so-called ‘social’ and the ‘biological’ – is to achieve any kind of epistemic force in the decades to come, then at least some of that force may come via recourse to a form of knowledge production that is, in fact, already aware of the potency of these exchanges: cognitive neuroscientific experiments.

## Experimental Entanglements

### Experiment: Entangled

At least since Ian Hacking’s *Representing and Intervening* (1983), scholars have addressed experiment and experimentation as complex, knowledge-producing phenomena in their own right, rather than simple accomplices of scientific theory (cf. Galison, 1987; Gooding et al., 1989; Davies, 2010). Some of the most compelling research in the history of science has indicated that if we want to understand, or, indeed, help foment, the formation of new knowledge-practices, we should not – as much discourse under the ‘regime of the inter’ does – focus our gaze at the scale of disciplines or paradigms. Rather, we should, as the historian of science Hans-Jörg Rheinberger has demonstrated in his work on modern experimental systems, be alert to:

the digressions and transgressions of smaller research units below the level of disciplines, in which knowledge has not yet become labeled and classified, and in which new forms of knowledge can take shape at any time … novelties generated in one system can quickly spread and create effects at other places. (2011: 315)

With Rheinberger, we direct attention to spaces of experimentation in which the intersections between scientific ‘objects’, instruments, apparatuses and experimenters still quiver with uncertainty – where the liveliness of experimentation has not yet been stilled by epistemological resolution. A living experimental system, Rheinberger argues, has ‘*more stories* to tell than the experimenter at a given moment is trying to tell with it’ (1994: 77–8). Because such a system still holds ‘excess’ within itself, it ‘contain[s] remnants of older narratives as well as fragments of narratives that have not yet been told’ (p. 78).

This account of excess underpins our argument for turning to experiment in cognitive neuroscience. One of the distinguishing characteristics of the contemporary neurosciences is that, because of the still-recent emergence of novel methods and sub-disciplines affiliated to this area, as well as their ongoing shuffling and realignment, core methods and assumptions have still not been entirely ossified (Abi-Rached, 2008). Certainly, this is subject to change, and some procedures and constructs – for example the relation between the BOLD (Blood Oxygenation Level Dependent) signal, which fMRI picks up, and brain activity – have over time been ‘black-boxed’ in a Latourian (1999) sense. But our collaborations with neuroscientists have consistently thrown up instances in which our collaborators were already deeply preoccupied with which of many methods to employ, how best to instruct research subjects, how to understand the relation between subject and researcher, how to operationalize constructs (e.g. Filevich et al., 2013), and so on. Cognitive neuroscience is thus a field in which many experimental systems are (still) in motion (e.g. Le Bihan et al., 2001, *Neurocritic*, 2012; Callard and Margulies, 2011). It is not a desire for control that undergirds our positive turn to experiment. Quite the opposite: we are compelled by the promise of digressions, transgressions, mistakes and the subterranean existence of not-as-yet-played-out narratives.

A core goal of ‘experimental entanglements’ is to intensify the energies *already* within these experimental systems by seeding research projects and centres with researchers carrying heterogeneous modes of practice from the social sciences and humanities. We wish to do so because we want to *magnify* the productive untidiness, and temporal out-of-jointedness of those systems. An expansion in styles of taking measurements, using instruments, engaging with research subjects and tinkering with protocols might just help both to render and expose new biosocial stories (Rheinberger, 2010: 218–19). Of course we are not naïve about how unevenly epistemic and institutional authority is likely to be distributed across such entanglements, and we do not elide the unequal dynamics of power and prestige here. Nor do we pretend that the desire to rethink paradigms, and to tinker with protocols, is likely to be as strong for neuroscientists *en masse* as it might well be for collaborating social scientists. We have no fantasy of parity here – nor do we assume that the most congenial and democratic spaces are always the most interesting or productive (Fitzgerald et al., 2014). We remain sanguine – we have no choice to act otherwise – about the likelihood of an experimental entanglement resulting in entropy, frustration, or failure.

**Figure 1.**
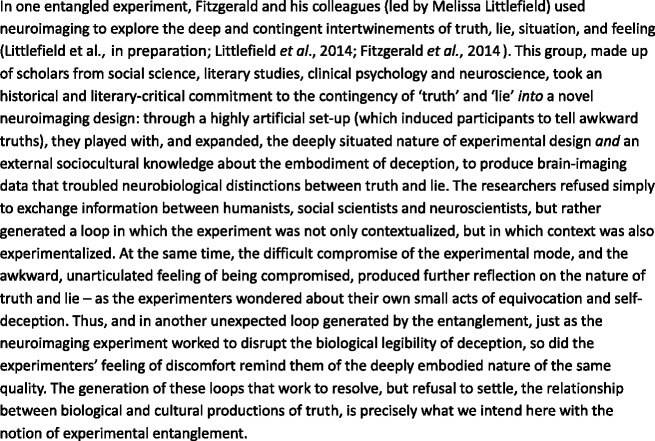
The neural correlates of deception: Imaging, history, context and feeling.

‘Experimental entanglements’ are modest, often awkward, typically unequal encounters that work to mobilize specific and often serendipitous moments of potential novelty in and outside the laboratory. These moments might reside in the methods chosen, the conduct of the experiment itself, the theoretical armature that surrounds it – or the roles that researchers play within the experiment, its analysis, and in its dissemination. ‘Experimental entanglements’ refuse preliminary decisions about the shape or outcome of such an interaction: they denote an ad hoc process of shuffling histories, methods, and assumptions from the social sciences and humanities *through* such partial moments, and of picking through the scraps of knowledge and thought produced *by* the subsequent torsion. Our ‘entanglements’ are thus *never not* temporary, local assemblages of motivation, interest, people and machinery – in which we, and our collaborators, are able momentarily to think something exterior to both the conventions of experimental practice, *and* the taken-for-granted dynamics of epistemic power that underwrite its conduct. This vision of being entangled is something very different from calls for neuroscientists to develop ‘second-order observations of laboratory conditions, communities of scientists, and historical and cultural contingencies’ (Slaby and Choudhury, 2012: 42). Our model, through its attention to untidiness, excess and chance, strives to avoid such pre-determined demands for reflexive practice from either side. Instead, we seek the entanglement of researchers, instruments, writing practices, discourses, observations, archives, bodies, topologies, and, in general, accounts of what that opaque object of neuroscientific research, around which all of these circle, just might be (Figure 1).

On such a model, *our own* knowledge practices will also, of course, be bound up with specific entanglements of context, thought and affect. Attending to experiment demands attending to how the bodies, gestures and feelings of individual researchers are registers and generators of positive knowledge. Natasha Myers, in her ethnography of experimental manoeuvres within molecular biology, has described how scientists’ bodily contortions can help to ‘render’ the objects of research; using the body, she argues, ‘can generate both new forms of knowing, and the things known’ (Myers, 2012: 172, 161; cf. Fitzgerald, 2013). We draw particular attention to this quality because one of the most potentially fertile attributes of many cognitive neuroscientific experiments is the dynamism enabled by the fact that there are commonly *at least* two minds and bodies – that of the experimenter and that of the experimental subject – built into the experimental assemblage. What we might call ‘the inter-subjective’ is *always already* instantiated in both the practice and the data of cognitive neuroscience – although this is rarely explicitly recognized in canonical texts (see e.g. Frackowiak et al., 2004; cf. Schilbach et al., 2013). Such entanglements pose multiple trajectories for novel inquiry: who or what is the instrument? Who or what probes whom or what? Who or what yields data? How are relations of influence and connection between experimenter and experimental subject imagined, materialized, felt, and traced out? Such combinatorial possibilities offer germs through which new forms of knowledge might emerge.

### Entanglement: Experimentalized

We have argued that it is increasingly difficult for the social sciences to maintain a potent hold on the expansive category of ‘human life’ while remaining indifferent to the complex neurogenetic textures of human capability. But while there is good reason, then, to cease the hygienic practices of many of the mainstream ‘social’ sciences (Goodman, 2013), no new epistemic model has yet emerged to express this possibility. In promoting a return to experiment, we contend that the laboratory spaces of the new brain sciences offer hitherto under-used fora to draw out the tangled biological and sociocultural processes of human life. We situate the cognitive neuroscientific experiment – understood as a tumbling and uncertain mode of knowledge-production – as one possible space in which both to register and to interpret these processes.

We draw inspiration from the work of feminist philosopher Karen Barad (2007) and her insight that sustainable and more-or-less bounded ways of producing knowledge might in fact come *after* – and not before – awkward mixtures of knowledge and material. Two features of Barad’s recent work give energy to our proposal. First, her account of an ‘agential realism’ attempts to think a constitutive relationship between the mess and ambiguity of entanglement, *and* the confounding possibility of distinction or singularity – with the latter coming after entanglement, and not before. Thus Barad’s approach:

does not take separateness to be an inherent feature of how the world is. But neither does it denigrate separateness as mere illusion … relations do not follow *relata*, but the other way around. (2007: 136–7)

Barad argues instead for a metaphysics based on ‘phenomena’ – a term that designates *both* ‘the ontological inseparability/entanglement of intra-acting agencies’ and the ‘primary ontological units’ of the world (Barad, 2007: 139–41; cf. Marres, 2012). That the inseparability of agencies does not mitigate against ‘determinate boundaries and properties of “entities” within phenomena’ is crucial for our account of ‘experimental entanglement’ (Barad, 2007: 148). Perhaps counter-intuitively, our approach wishes to similarly preserve *both* the fundamental inseparability of the biological and the sociocultural, *and* the possibility of a subsequent cut. If we refuse to position neuroscientific experiments as bounded or controlled spaces, we do not regard them as doomed to a morass of uncertainty. While we wish to affirm the ontological and methodological ‘mess’ of any neuroscientific experiment, we also contend that such experiments are able to produce meaningful knowledge about the biosocial complexities of human life.

Second, Barad refuses to separate the practice of science from the practice of studying science from the outside: ‘the tradition in science studies’, she points out, ‘is to position oneself at some remove, to reflect on the nature of scientific practice as a spectator’ (2007: 247). Barad invites us instead to think about the ways in which insights about the so-called ‘social context’ of science might also be intrinsic to the scientific practices in question (2007: 247). She posits a mode of engagement in which an ‘understanding of the entangled co-emergence of “social” and “natural” (and other important co-constituted) factors might best come from ‘engaging in practices we call “science studies” together with practices we call “science”’ (2011: 446).

With these two interventions, Barad proposes a radically different programme for sociocultural attention to, and ‘engagement with’, the natural sciences. In particular, she departs from modes of interdisciplinary engagement, which, as with all modes governed by the ‘regime of the inter-’, are premised on a recognition of the *solidity* of disciplinary borderlands (however deeply either envisages trade and exchange across those boundaries; see Galison, 1997; Thompson Klein, 2010). Our ‘experimental entanglements’ follow Barad in their insistence that neurobiological knowledge is a *product of*, and not a *precursor to*, disciplinary transaction – that the complex intersections of social and biological agencies come *prior* to, for example, the kind of agential cut that critical neuroscience insists on maintaining. Indeed, in a formal sense, introducing ‘critique’ and ‘context’ really does pollute the neuroscientific experiment – but precisely because this insistence *reduces* entangled complexity to a series of distinctive and competing perspectives.

**Figure 2.**
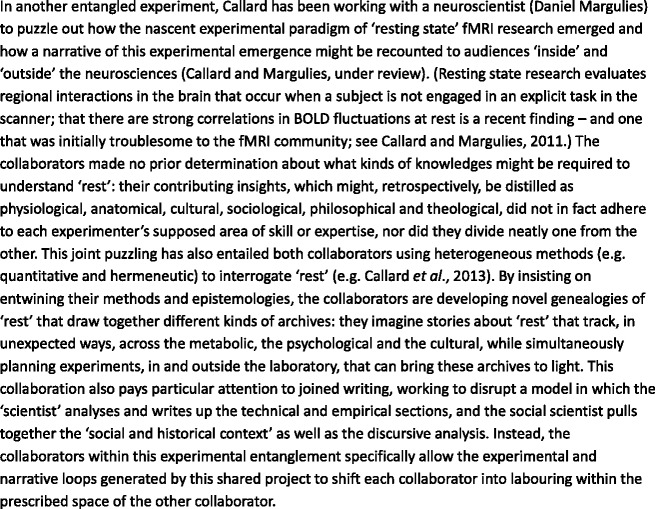
Experimenting with ‘rest’ in fMRI research.

There are costs to taking this position seriously – as we do. In particular, because Barad’s conception of entanglement insists on the ontological *priority* of intersection, it becomes methodologically fruitless, in the kinds of experiment we envisage, to delineate distinct tasks, inputs and divisions of labour for ‘social scientists’ and ‘neuroscientists’ in advance. It is not a commitment to obscurantism that makes us resistant to clearly setting out, for example, ‘who’ might do ‘what’ within an ‘experimental entanglement’. Rather, we maintain that ideas about ‘who’ and ‘what’ must remain in play when we proceed on the assumption that entanglements – of bodies, epistemologies, apparatuses, elements of experimental systems, operationalizations of terms – might produce something new in the world, even as the forms that that newness might take are undecided, and undecidable, prior to the moment of experimentation (for example, see Figure 2).

We are insistent that this suggestion is not opposed to the ethic and ethos of experiment as such (e.g. see Donna Haraway’s (1997) ‘modest’ modes of engaging experimental spaces). Roepstorff and Frith, for example, in their reflections on neuroanthropology, direct attention to the experimental itself, as a productive object to think with: experimentalism, they point out, is ‘a complicated practice, a *bricolage* tinkering with the possible elements (Pickering, 1995) to make things work’ (2012: 103). Thus, experiments do not – and are not supposed to – *settle matters*: an experiment in neuroimaging, no less than the much-analysed space of anthropological fieldwork, is variously intimate, awkward, lonely and boring; the generation of facts from data, in the neuroscientific laboratory, has never not been painful, messy, unsatisfying, and contingent. With this in mind, Roepstorff and Frith encourage us to regard the ‘experiment’ not as a nitty-gritty, world-testing, fact-producing machine, but as a *performance* – and thus potentially as a risky, more avant-garde space. They describe this as an ‘aesthetics of research practice’, a mode of engagement in which the neuroimaging experiment becomes something akin to ‘trying out new ways of writing, new ways of being in the field, or novel forms of intervention’ (2012: 105). And they suggest that it is a form of aesthetic attention that allows the social scientist to take some kind of experimental rubric into her fieldwork. We linger on this description because we, too, are committed to using the experimental mode to rethink the ways in which relations across the social sciences and neurosciences are imagined and materialized. Our aesthetics of experiment fixes attention on the capacity of experimental intervention to unfold, to ally itself with, and then to elaborate upon, the determinedly entangled nature of human subjectivity.

## Conclusion

We asked, at the start of this article, what might happen if we set aside our usual disciplinary allegiances and identifications to think more experimentally about the constitution and dynamics of the cognitive-neuroscientific-experimental domain. Our question was driven by our weariness with what we have described as the ‘regime of the inter-’: a regime which, we believe, has not only too frequently resulted in social scientists either clapping or barking at the neurosciences, but has commandeered both the imaginative and institutional space through which engagements ‘between’ the social sciences and the neurosciences might be envisaged. Our urge to disrupt this regime is motivated by our desire to move beyond the etiolated and benumbed visions of experiment and experimentation that, too commonly, are proffered under it.

The founding principle of an experimental entanglement is that it is ‘discipline’ that needs explanation, not promiscuity. What might be imagined as a securely ‘cultural’ or ‘social’ knowledge is a *product* of collaboration with the biological (and other) sciences: it is not a precursor to that collaboration. Our use of the term entanglement thus signals our growing suspicion that the central epistemological and institutional problem is not one of whether, or to what degree, disciplinary and epistemic boundaries might be crossed. The pressing question, it seems to us, is how, as human scientists, we are to produce knowledge amid a growing realization that those boundaries are pasted across objects which are quite indifferent to a bureaucratic division between disciplines; and that scholars and researchers of all stripes invariably attend to, and live among, objects whose emergence, growth, development, action, and disappearance do not at all admit of neat cuts between the biological and the social, or between the cerebral and the cultural.

The labours of experimentation are frequently onerous and fruitless. And this has been as evident to some scholars in the humanities and social scientists – where there is also, of course, a rich legacy of experiment and experimentation (e.g. Clifford and Marcus, 1986; Clough, 2000) – as it doubtless is to many practising cognitive neuroscientists. But those labours can also yield unexpected harvests. We have argued that the cognitive neuroscientific experiment – understood as a kind of narrative excess, interpreted as an aesthetics, and approached with intellectual modesty – might be a space in which richer elaborations of human subjectivity might materialize than is commonly imagined. This is not a demand that the sociocultural and interpretative sciences ‘reduce’ themselves to the manipulation of laboratory apparatuses: ours is not a fantasy in which hordes of social scientists are re-directed from libraries and offices to the neuroimaging scanners in the basements. But it *is* a call for a more expansive imaginary of what experiment – as practice and ethos – offers to practitioners within those disciplines. Our suggestion is that it might offer a moment in which some elements of the biosocial entanglement of human life are centrally at stake, and in which they might be brought into some kind of richer understanding. We have many more suggestions for what those moments might look like in practice (Callard and Fitzgerald, under contract). This article establishes some of the core theoretical ground for our having made that move; it must end as an invitation to the interested reader to step outside the ‘regime of the inter-’ and begin to trace her own trajectories of entanglement.

## Notes

This article was completed while DF was funded by an Interacting Minds Centre project on Neuroscientific Evidence, at Aarhus University (Denmark), and by an ESRC (UK) Transformative grant on ‘A New Sociology for a New Century’ (ES/L003074/1). FC’s research for this article has been supported by two Wellcome Trust Strategic Awards to Durham University (WT086049 and WT098455MA). Both DF and FC also gratefully acknowledge an award from the Volkswagen Foundation’s Second European Platform for Life Sciences, Mind Sciences and Humanities, which funded a workshop on ‘Experimental Entanglements in Cognitive Neuroscience’.

1 Note that ‘webs of human social and cultural life’ have been figured quantitatively and ‘scientifically’ in several social science disciplines for some time – not least in the archaeological and geographical sciences. Here, however, we address ourselves to those parts of the social sciences and humanities whose intellectual roots are in the emergence of the ‘social’ and/or ‘cultural’ as a distinct object of knowledge, and within which, tentative, empirically-focused turns towards biology have not been met with alacrity. See Donzelot (1988), Rose (1991), and Latour (2005).

2 Historians of science, sociologists and researchers in science and technology studies (STS) have taken ‘experimental labour’ as an object of study; we want here to explore how cognitive neuroscientific experimentation might be a methodological and epistemological resource for social scientists and humanists. We are indebted to (and expand upon) some recent exceptions to the general disregard for this question, such as Nikoleyczik’s ‘multidimensional’ and ‘integrative’ approach (2012); see also Bluhm et al. (2012), and Roepstorff and Frith’s (2012) ethic of conceptual ‘front-loading’.

3 This article draws on our many years of separate and conjoined engagement with interdisciplinary neurobiological-sociocultural experimentation. What we here name as ‘experimental entanglement’ theorizes our longstanding frustration with the ‘interdisciplinary’ approaches that dominated these engagements. Here, we articulate the conceptual ground that lies beneath this frustration; more detailed case analyses of some of the ‘entanglements’ that we have helped to initiate are provided in Callard and Fitzgerald (under contract).

4 In this article, we move between the ‘neurosciences’ and ‘cognitive neuroscience’. The neurosciences incorporate a huge range of methods and foci that encompass molecular, cellular, developmental, structural, functional, evolutionary and computational studies of the brain in its ‘normal’ and ‘abnormal’ states (see Rees and Rose, 2004, or Abi-Rached, 2008). It is most commonly cognitive neuroscience that is the focus of attention in the social sciences and humanities.

5 Some social scientific (and humanist) research, in approaching the neurosciences as an object of historical and/or sociological study, does not neatly fall into any of the three modes we delineate below. In this article, we are interested in social scientific scholarship that does not simply take the neurosciences as an object of study, but rather addresses how the growth in the neurosciences poses questions vis-à-vis how the social sciences might or should respond to this.

6 The concept of critique of course has great semantic density as well as a complex genealogy, as De Boer and Sonderegger (2012) demonstrate.

7 Some deflationary accounts leave open space for what they think might be more productive ‘interdisciplinary ventures between the humanities and the sciences’ (Kramnick, 2011), but they tend, overall, not to be interested in the mechanics of such ventures.

8 Slaby and Choudhury argue, specifically, that: While critical neuroscience does not directly follow a Frankfurt School program … it does share with it a spirit of historico-political mission; that is, the persuasion that scientific inquiry into human reality tends to mobilize specific values and often works in the service of interests that can easily shape construals of nature or naturalness. These notions of nature or of what counts as natural … require unpacking. Without critical reflection, they appear as inevitable givens, universal and below history, and are often seen as a form of ‘normative facticity,’ making specific claims upon us in everyday life. (2012: 29)

9 Of course, the interactive mode, too, has a history – not least a history of transdisciplinary scholars, or those working in formative moments for their disciplines, who thought the experimental relationship between social life, psychological life, and the brain. Particularly noteworthy here are the works of, for example, Kurt Lewin (1947) and Kurt Goldstein (2000 [1939]).

10 For example, see the concluding comments of Ashton (2011), a literary theorist, in her critique of neuroaesthetics: This essay argues for why we should not just be delighted with the [neuroaesthetic] results, or rather, why we can’t be delighted with the results and still maintain a coherent account of what we’re doing when we’re doing the interpretive work of literary or art history and criticism …. Neuroaesthetics is answering a set of questions about causes, while the interpretation of a work of art depends on having answers about its meaning.

11 See, for example, documents on the European Commission’s unfolding ‘Horizon 2020’ research and innovation programme, which argues that: Radical breakthroughs with a transformative impact increasingly rely on intense collaboration across disciplines in science and technology (for instance, information and communication, biology, chemistry, earth system sciences, material sciences, neuro- and cognitive sciences, social sciences or economics) and with the arts and humanities. This requires not only excellence in science and technology but also new attitudes and novel interactions between a broad range of players in research. (European Commission, 2011: 35).
